# A Quorum Sensing-Disrupting Brominated Thiophenone with a Promising Therapeutic Potential to Treat Luminescent Vibriosis

**DOI:** 10.1371/journal.pone.0041788

**Published:** 2012-07-25

**Authors:** Tom Defoirdt, Tore Benneche, Gilles Brackman, Tom Coenye, Patrick Sorgeloos, Anne Aamdal Scheie

**Affiliations:** 1 Laboratory of Aquaculture & Artemia Reference Center, Ghent University, Ghent, Belgium; 2 Department of Chemistry, Faculty of Chemistry, University of Oslo, Oslo, Norway; 3 Laboratory of Pharmaceutical Microbiology, Ghent University, Ghent, Belgium; 4 Department of Oral Biology, Faculty of Dentistry, University of Oslo, Oslo, Norway; The Scripps Research Institute, United States of America

## Abstract

*Vibrio harveyi* is amongst the most important bacterial pathogens in aquaculture. Novel methods to control this pathogen are needed since many strains have acquired resistance to antibiotics. We previously showed that quorum sensing-disrupting furanones are able to protect brine shrimp larvae against vibriosis. However, a major problem of these compounds is that they are toxic toward higher organisms and therefore, they are not safe to be used in aquaculture. The synthesis of brominated thiophenones, sulphur analogues of the quorum sensing-disrupting furanones, has recently been reported. In the present study, we report that these compounds block quorum sensing in *V. harveyi* at concentrations in the low micromolar range. Bioluminescence experiments with *V. harveyi* quorum sensing mutants and a fluorescence anisotropy assay indicated that the compounds disrupt quorum sensing in this bacterium by decreasing the ability of the quorum sensing master regulator LuxR to bind to its target promoter DNA. *In vivo* challenge tests with gnotobiotic brine shrimp larvae showed that thiophenone compound TF310, (*Z*)-4-((5-(bromomethylene)-2-oxo-2,5-dihydrothiophen-3-yl)methoxy)-4-oxobutanoic acid, completely protected the larvae from *V. harveyi* BB120 when dosed to the culture water at 2.5 µM or more, whereas severe toxicity was only observed at 250 µM. This makes TF310 showing the highest therapeutic index of all quorum sensing-disrupting compounds tested thus far in our brine shrimp model system.

## Introduction

*Vibrio harveyi*, the causative agent of luminescent vibriosis, is one of the most important pathogens of aquatic animals, causing significant losses in the aquaculture industry worldwide [1]. Because of the development and spread of antibiotic resistance in these bacteria, antibiotic treatments are becoming inefficient and therefore, alternative control strategies are being developed [2]. One of these strategies is the disruption of quorum sensing, bacterial cell-to-cell communication.

*V. harveyi* is one of the model bacteria in studies on quorum sensing. The bacterium contains a three-channel quorum sensing system, with three different types of signal molecules (HAI-1, AI-2 and CAI-1, respectively) feeding a common signal transduction cascade). In addition to bioluminescence, *V. harveyi* quorum sensing has been found to control biofilm formation [3] and the expression of different virulence factors, including a type III secretion system [4], extracellular toxin [5], metalloprotease [6], siderophore [7], chitinase [8] and phospholipase [9]. Moreover, we found that virulence of the bacterium towards different aquatic organisms, including brine shrimp, is also regulated by its quorum sensing system [10].

Because quorum sensing regulates virulence gene expression of different bacteria that are pathogenic towards plants, animals and humans, many research groups have investigated the synthesis and use of small molecules to disrupt quorum sensing-regulated virulence gene expression (for reviews see [11]–[13]). Halogenated furanones are amongst the most intensively studied quorum sensing-disrupting compounds. These compounds were found to disrupt the expression of quorum sensing-regulated genes in *V. harveyi* by decreasing the DNA-binding activity of the quorum sensing master regulator LuxR [14]. The application of quorum sensing-disrupting halogenated furanones has been reported to significantly decrease the virulence of different vibrios (including *V. anguillarum, V. campbellii, V. harveyi* and *V. parahaemolyticus*) towards different aquatic hosts [15]–[17]. However, a major problem of these compounds is their relative high toxicity towards higher organisms, with a ratio of 2.5–4 between toxic and therapeutic concentrations in our brine shrimp model system [16]. This is a major constraint with respect to practical applications, especially when dealing with very sensitive animals (e.g. aquatic larvae).

**Figure 1:**
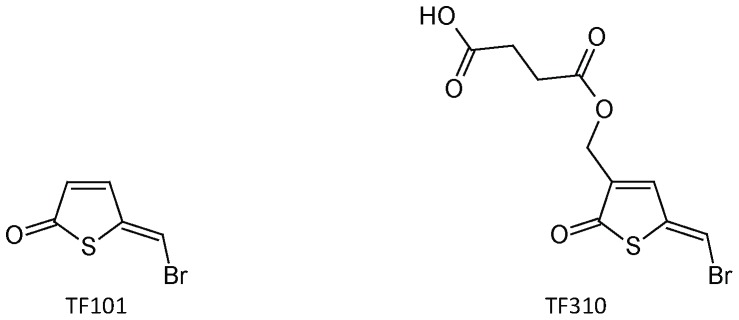
Structure of the brominated thiophenones used in this study.

In search for novel quorum sensing-disrupting compounds, Benneche and coworkers recently reported the synthesis of brominated thiophenones, sulphur analogues of the brominated furanones [18]. Interestingly, (*Z*)-5-(bromomethylene)thiophen-2(5*H*)-one (TF101; **Figure 1**) was more active with respect to inhibition of biofilm formation in *Staphylococcus epidermidis* [19] and *V. harveyi* [18] than the corresponding furanone. In the present study, we aim at investigating the molecular mechanism by which brominated thiophenones inhibit quorum sensing in *V. harveyi* and at evaluating their capacity to protect brine shrimp larvae from luminescent vibriosis. We used TF101 and (*Z*)-4-((5-(bromomethylene)-2-oxo-2,5-dihydrothiophen-3-yl)methoxy)-4-oxo-butanoic acid (TF310; **Figure 1**) [18]–[20], as test compounds.

## Results

### Thiophenones TF101 and TF310 block quorum sensing-regulated bioluminescence of V. harveyi wild type and quorum sensing mutants

**Figure 2:**
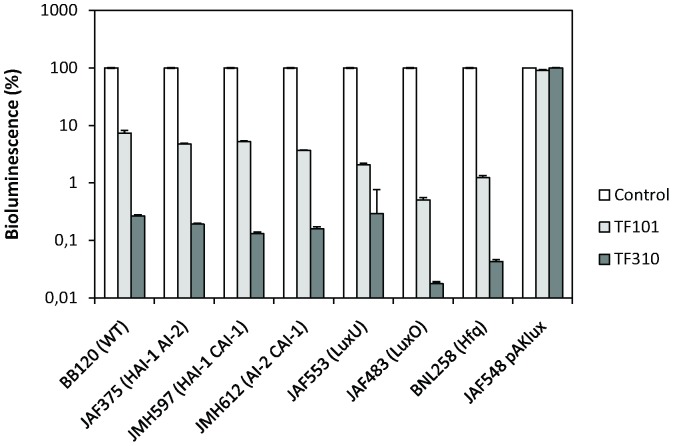
Bioluminescence of the *V. harveyi* wild type (BB120), the double mutants JAF375 (sensor HAI-1^−^, sensor AI-2^−^, sensor CAI-1^+^), JMH597 (sensor HAI-1^−^, sensor AI-2^+^, sensor CAI-1^−^) and JMH612 (sensor HAI-1^+^, sensor AI-2^−^, sensor CAI-1^−^), the constitutively luminescent quorum sensing signal transduction cascade mutants JAF553 (LuxU), JAF483 (LuxO) and BNL258 (Hfq), and strain JAF548 pAKlux1 (in which bioluminescence is independent of the quorum sensing system), with and without the thiophenones (2.5 µM). Luminescence measurements were performed 0.5 h after the addition of the thiophenones. For each strain, the bioluminescence without the addition of thiophenone was set at 100% and the other samples were normalised accordingly. The error bars represent the standard deviation of four replicates.

Bioluminescence is one of the phenotypes that is regulated by the *V. harveyi* quorum sensing system and therefore, in a first experiment, the impact of the thiophenones on the bioluminescence of *V. harveyi* was determined. Wild type strain BB120 was grown to high cell density in order to activate quorum sensing-regulated bioluminescence, after which the thiophenones were added to the medium at 2.5 µM. Bioluminescence was measured 0.5 h after the addition of the thiophenones and both compounds were found to block bioluminescence (**Figure 2**).

Further, in order to determine the impact of the compounds on the three different channels of the quorum sensing system, the signal molecule receptor double mutants JAF375 (sensor HAI-1^−^, sensor AI-2^−^, sensor CAI-1^+^), JMH597 (sensor HAI-1^−^, sensor AI-2^+^, sensor CAI-1^−^) and JMH612 (sensor HAI-1^+^, sensor AI-2^−^, sensor CAI-1^−^) were used. Because of the inactivated receptors, bioluminescence in these mutants is only responsive to one of the three signal molecules [21]. Bioluminescence was found to be blocked by the thiophenones in all three double mutants (**Figure 2**), indicating that all three channels of the quorum sensing system were blocked.

Finally, the effects of the thiophenones on bioluminescence of quorum sensing signal transduction cascade mutants were investigated. The mutants JAF553 and JAF483 contain a point mutation in the *luxU* and *luxO* genes, respectively, rendering the LuxU and LuxO proteins incapable of phosphorelay [22], [23]. Strain BNL258 has a Tn5 insertion in the *hfq* gene, resulting in a non-functional Hfq protein [24]. Hence, because of the nature of these mutations, the three mutants are constitutively luminescent and therefore, blocking luminescence in one of them would indicate that the thiophenones act downstream of the mutated component. The compounds blocked luminescence in all three mutants (**Figure 2**), suggesting that they act downstream of Hfq, i.e. at the level of the quorum sensing master regulator LuxR.

**Figure 3:**
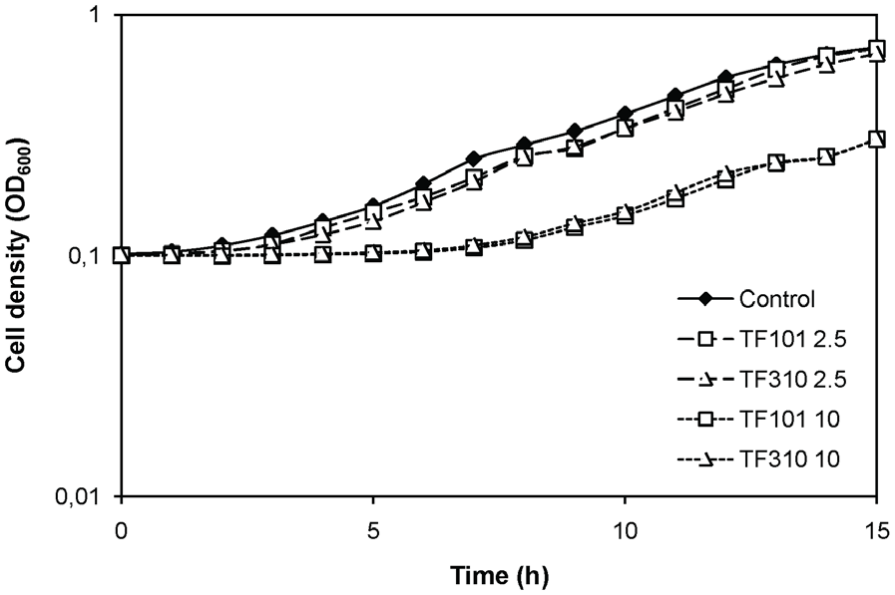
Growth of *Vibrio harveyi* BB120 in Luria-Bertani medium containing 35 g l^−1^ of synthetic sea salt, with and without the thiophenones at 2.5 and 10 µM (average of 4 replicates). Error bars are too small to be visible.

Importantly, at 2.5 µM, the compounds had no effect on the growth of *V. harveyi* (**Figure 3**). In order to exclude the possibility that the effect observed in the bioluminescence experiments was due to (minor) toxicity, we investigated the effect of the compounds on bioluminescence of a strain expressing the bioluminescence operon independently of the quorum sensing system. To this end we used the dark QS mutant JAF548, in which the QS signal transduction cascade mimicks the situation where no signal molecules are present and hence the native bioluminescence operon (which is under QS control) is not activated. We conjugated plasmid pAK*lux*1 [25] into this strain. The plasmid contains the *Photorhabdus luminescens* bioluminescence operon under the control of a constitutive promoter. Hence, strain JAF548 pAK*lux*1 produces bio luminescence that is independent of the quorum sensing system. We found that 2.5 µM of TF101 had a small (but significant; P<0.05) effect on bioluminescence of JAF548 pAK*lux*1, whereas TF310 had no effect (**Figure 2**).

### Thiophenones TF101 and TF310 inhibit binding of LuxR to target promoter DNA

**Figure 4:**
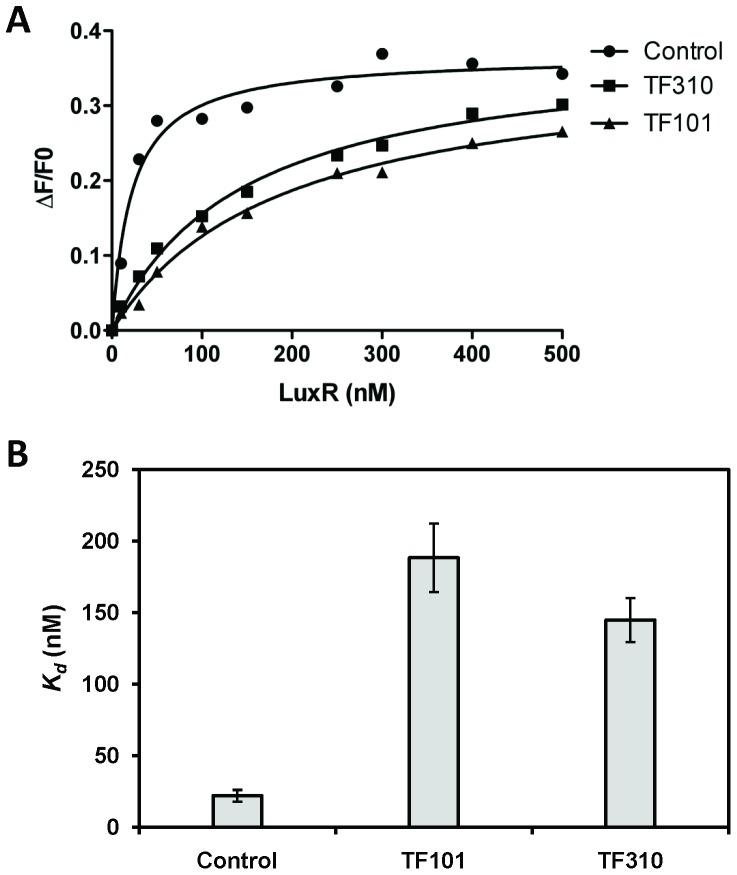
LuxR DNA binding with and without the thiophenones (10 µM). **A.** The fractional change in anisotropy, ΔF/F_o_, is plotted against the concentration of purified LuxR. **B.** *K_d_* values are calculated as the half-maximal fractional change in fluorescence anisotropy.

To investigate the effect of the thiophenones on the DNA-binding activity of the quorum sensing master regulator LuxR, a fluorescently labelled fragment of a *V. harveyi* consensus LuxR binding sequence was incubated together with purified LuxR, with and without the thiophenones (10 µM). Incubation of LuxR together with the fragment resulted in a significant increase in anisotropy (**Figure 4**). Addition of the thiophenones strongly blocked the increase in anisotropy, indicating that the compounds reduce the DNA-binding activity of LuxR.

### Thiophenone TF310 protects brine shrimp larvae from luminescent vibriosis

We previously showed that the virulence of *V. harveyi* BB120 in our gnotobiotic brine shrimp model system is regulated by quorum sensing [10]. As both thiophenone compounds were found to block quorum sensing in *V. harveyi*, we investigated whether the compounds could protect brine shrimp larvae from the pathogen in *in vivo* challenge tests. Thiophenone TF101 was highly toxic since we observed almost complete mortality of brine shrimp larvae at concentrations of 5 µM or higher, whereas no protection was observed at 1 µM of TF101 (survival of challenged larvae treated with 1 µM of TF101 was not significantly different from that of untreated challenged larvae; data not shown). On the other hand, thiophenone TF310 increased the survival of challenged larvae when added to the culture water at 1 µM or more, offering a complete protection (no significant difference in survival with non-challenged larvae) at concentrations of 2.5 µM or higher (**Table 1**). Thiophenone TF310 was much less toxic than compound TF101 since no significant mortality occurred in larvae exposed to 50 µM of TF310. At 100 µM, there was significant mortality, although the survival was still high (79%), while at 250 µM, complete mortality was observed (**Table 1**).

**Table 1:** Relative percentage survival^a^ of brine shrimp larvae with and without TF310 (average ± standard deviation of three replicates), after 2 days, with and without the pathogen *V. harveyi* BB120.

Treatment	Survival^a^ (%)		
Control	100	±	3 ^A^
TF310 5 µM	95	±	3 ^A^
TF310 20 µM	95	±	8 ^A^
TF310 50 µM	91	±	3 ^A,B^
TF310 100 µM	79	±	8 ^B,C^
TF310 250 µM	0	±	0 ^E^
BB120	46	±	6 ^D^
BB120+TF310 1 µM	64	±	14 ^C^
BB120+TF310 2.5 µM	108	±	9 ^A^
BB120+TF310 5 µM	96	±	8 ^A^
BB120+TF310 20 µM	96	±	6 ^A^

a Survival in the control treatment was set at 100% and the other treatments were normalised accordingly. Treatments with a different superscript letter are significantly different from each other (ANOVA with Duncan post-hoc test; P<0.01).

## Discussion

Brominated thiophenones, sulphur analogues of the well-known quorum sensing inhibiting brominated furanones, were recently reported to block biofilm formation in *V. harveyi* [18]. In the present study, we aimed at investigating the mechanism by which the brominated thiophenones TF101 and TF310 inhibit quorum sensing in *V. harveyi* and at evaluating their potential to protect brine shrimp larvae from luminescent vibriosis.

We previously reported that brominated furanones inhibit quorum sensing in *V. harveyi* by decreasing the DNA-binding activity of the quorum sensing master regulator LuxR, which is located at the end of the quorum sensing signal transduction cascade [14]. As the thiophenones are structurally highly similar to brominated furanones, we hypothesised that they would have a similar mode of action. Indeed, the compounds also blocked bioluminescence of *hfq* mutant BNL258. Hfq is a chaperone protein that acts together with small regulatory RNAs to destabilise the mRNA of the quorum sensing master regulator LuxR. The Hfq protein is non-functional in strain BNL258, resulting in constitutively expressed bioluminescence [24]. The fact that the thiophenones blocked bioluminescence in this mutant indicated that they act downstream of Hfq, i.e. at the level of LuxR.

**Figure 5:**
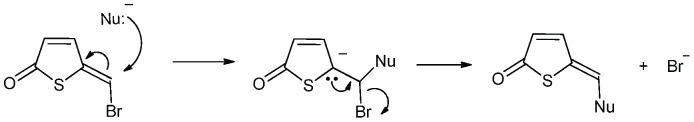
Proposed reaction mechanism of the thiophenones. Nu: nucleophile. Candidate nucleophiles are e.g. thiol groups of cysteine residues.

A fluorescence anisotropy assay with a labelled LuxR target sequence indicated that both compounds decrease the DNA-binding activity of LuxR. Interestingly, Zang and coworkers reported that brominated furanones covalently bind to a cysteine residue in the LuxS enzyme, thereby inactivating the enzyme [26]. Based on our data, we hypothesise that the thiophenones bind to one or more nucleophilic amino acid residues in LuxR by a similar addition-elimination mechanism (**Figure 5**). We have recently shown that the 5-bromomethylene side-chain of TF101 reacts with thiols and amines under basic conditions by an addition-elimination mechanism [27]. Candidate nucleophilic amino acid residues include 4 cysteine residues in the C-terminal dimerisation domain of LuxR [28]. Binding to one of these residues would likely decrease the ability of LuxR to form a dimer, thereby decreasing the ability to bind to target promoter DNA (LuxR is a member of the TetR family of transcriptional regulators, which bind DNA as dimers [29]). However, further research is needed to confirm this hypothesis.

*In vivo* challenge experiments with gnotobiotic brine shrimp larvae revealed that thiophenone TF310 increased the survival of larvae challenged to pathogenic *V. harveyi* when added to the culture water at 1 µM or more, offering a complete protection (no significant difference in survival with non-challenged larvae) at concentrations of 2.5 µM or higher. Together with the *in vitro* results, this indicates that thiophenones are more active than the corresponding brominated furanones, with a concentration of 2.5 µM having a similar effect as ∼100 µM of the furanones [14], [16].

The *in vivo* experiments also revealed that compound TF101 was highly toxic to the brine shrimp, whereas TF310 was not. In contrast, both compounds had similar quorum sensing-disrupting activities. Hence, the side chain significantly decreases toxicity of the thiophenones, without affecting quorum sensing-disrupting activity. Similar results have been reported for brominated furanones, in which the presence of a 3-alkyl side chain resulted in lower toxicity to planktonic bacterial cells, without major impact on quorum sensing-disrupting activity [30], [31]. As brominated furanones and brominated thiophenones are highly reactive molecules, we hypothesise that inactivation of essential proteins (caused by binding of the compounds to nucleophilic amino acid residues), is responsible for their toxicity. The presence of a side chain possibly limits the access of the furanones and thiophenones to some nucleophilic amino acid residues of these proteins (due to steric hindrance), thereby reducing the toxicity of the compounds.

In conclusion, the results presented in this study showed that the brominated thiophenones TF101 and TF310 inhibit quorum sensing-regulated gene expression in *V. harveyi* by decreasing the DNA-binding activity of the quorum sensing master regulator LuxR. *In vivo* challenge tests with gnotobiotic brine shrimp larvae showed that compound TF310 efficiently protected the larvae from luminescent vibriosis, whereas compound TF101 was highly toxic. Thiophenone TF310 has the highest therapeutic index of all quorum sensing-disrupting compounds tested thus far in our brine shrimp model system (**Table 2**).

**Table 2:** Comparison of the therapeutic potential of different quorum sensing-disrupting compounds in the gnotobiotic brine shrimp - *V. harveyi* model.

Compound	Target	[QSI]_50_ a (µM)	[Therapeutic]^b^ (µM)	[Toxic]^c^ (µM)	References
Thiophenone TF310	LuxR	<2.5	2.5	250	This study
Brominated furanone C-2	LuxR	20	65	160	[16]
Cinnamaldehyde	LuxR	100	100	250	[32]
LMC-21	LuxPQ	20	40	>100	[33]

a Concentration needed to obtain 50% decrease in quorum sensing-regulated bioluminescence in *V. harveyi* BB120 *in vitro*.

b Concentration needed to completely protect gnotobiotic brine shrimp larvae from *V. harveyi* BB120.

c Concentration at which the compound causes high mortality in brine shrimp larvae.

## Materials and Methods

### Thiophenones

Thiophenone TF101, (*Z*)-5-(bromomethylene)thiophen-2(5*H*)-one (**Figure 1**), was synthesized as reported previously [34]. Thiophenone TF310, (*Z*)-4-((5-(bromomethylene)-2-oxo-2,5-dihydrothiophen-3-yl)methoxy)-4-oxobutanoic acid, was synthesized according to the scheme presented in **Information S1**. The thiophenones were dissolved in pure ethanol at 50 mM and stored at −20°C.

### Bacterial strains and growth conditions

*V. harveyi* strains used in this study are shown in **Table 3**. Strain JAF548 pAK*lux*1 was constructed by conjugating plasmid pAK*lux*1 into strain JAF548 as described before (Karsi et al., 2006). Unless otherwise indicated, all strains were grown in Luria-Bertani medium containing 35 g l^−1^ of Instant Ocean synthetic sea salt (Aquarium Systems Inc., Sarrebourg, France) at 28°C under constant agitation. Spectrophotometry at OD_600_ was used to measure growth.

**Table 3:** *V. harveyi* strains used in this study.

Strain	Phenotype	References
BB120	( = ATCC BAA-1116) wild type from which strains BNL258, JAF375, JAF483, JAF553, JAF548, JMH597 and JMH612 were derived	[35]
BNL258	*hfq*::Tn5*lacZ*	[24]
JAF375	*luxN*::Cm^R^ *luxQ*::Kan^R^	[22]
JAF483	*luxO* D47A linked to Kan^R^	[22]
JAF553	*luxU* H58A linked to Kan^R^	[23]
JAF548	*luxO* D47E linked to Kan^R^	[22]
JMH597	*luxN*::Tn5 *cqsS*:: Cm^R^	[21]
JMH612	*luxPQ*::Tn5 *cqsS*:: Cm^R^	[21]
JAF548 pAK*lux*1	Luminescence independent of the quorum sesning system	This study

### Bioluminescence assays

*V. harveyi* wild type and quorum sensing mutants were grown overnight and diluted to an OD_600_ of approximately 0.5. The thiophenones were added at 2.5 µM and the cultures were further incubated at 28°C with shaking. 0.5 h after thiophenone addition, luminescence was measured with a Tecan Infinite 200 microplate reader (Tecan, Mechelen, Belgium).

### LuxR DNA-binding assay

*E.coli* BL21 pGET-1 (containing a *gst-luxR* overexpression construct) [36] was grown in Luria-Bertani broth with aeration at 37°C. Induction of GST-LuxR overexpression and protein purification were conducted as described previously [36]. GST-LuxR was purified using Glutathione Uniflow resins (Clontech, Mountain view, CA, USA) and fractions containing GST-LuxR were identified by both SDS-PAGE and capillary electrophoresis (Experion PRO260 chip, Bio-rad laboratories, Nazareth, Belgium). 5′fluorescein labelled DNA oligonucleotide (TATTGATAAATTTATCAATAA) and its unlabelled complement were obtained from Sigma-Aldrich. Annealing of the complementary nucleotides was achieved by heating equimolar concentrations in NaCl-Tris-EDTA buffer at 94°C for 2 min, after which the reaction mixtures were allowed to slowly cool to room temperature. Fluorescence polarization measurements in the presence and absence of the thiophenones were conducted as described previously [37]. Samples were excited at 480 nm and emission was measured at 535 nm on a Perkin Elmer EnVision plate reader at 30°C. *K_d_* values were calculated as the concentration of LuxR at the half-maximal fractional change in fluorescence anisotropy and curves were fit by non-linear regression using the Graphpad software (Graphpad Software Inc., La Jolla, CA, USA).

### Axenic hatching of brine shrimp larvae

All experiments were performed with high quality hatching cysts of *Artemia franciscana* (EG® Type, INVE Aquaculture, Dendermonde, Belgium). 200 mg of cysts were hydrated in 18 ml of tap water for 1 h. Sterile cysts and larvae were obtained via decapsulation, adapted from [38]. Briefly, 660 µl of NaOH (32%) and 10 ml of NaOCl (50%) were added to the hydrated cyst suspension. The decapsulation was stopped after 2 min by adding 14 ml of Na_2_S_2_O_3_ (10 g l-1). The decapsulated cysts were washed with autoclaved artificial seawater containing 35 g l^−1^ of Instant Ocean synthetic sea salt (Aquarium Systems Inc., Sarrebourg, France). The cysts were resuspended in a 50 ml tube containing 30 ml of autoclaved artificial seawater and incubated for 24 h on a rotor (4 min^−1^) at 28°C with constant illumination (approximately 2000 lux). Cyst breaking and nauplius hatching occurred after about 12 hrs, respectively 16 hrs incubation.

### Brine shrimp challenge tests

The shrimp were cultured in groups of 20 larvae in glass tubes containing 10 ml synthetic sea water (35 g l^−1^ Instant Ocean). The larvae were fed an autoclaved suspension of *Aeromonas* sp. LVS3 bacteria at 10^7^ cells ml^−1^ and *V. harveyi* BB120 was added at 10^5^ CFU ml^−1^, as described previously [16]. The thiophenones were added directly to the culture water at different concentrations. Afterwards, the glass tubes were put back on the rotor and kept at 28°C. Brine shrimp cultures to which only autoclaved LVS3 bacteria were added, were used as controls. The survival of the larvae was scored 2 days after the addition of the pathogen. All manipulations were done under a laminar flow hood in order to maintain sterility of the cysts and larvae. Each treatment was done in triplicate.

### Statistics

ANOVA analysis with Duncan post-hoc test was performed using the SPSS software, version 19.

## Supporting Information

Information S1 **Synthesis of (Z)-4-((5-(bromomethylene)-2-oxo-2,5-dihydrothiophen-3-yl)methoxy)-4-oxobutanoic acid (TF310).**
(DOC)
