# Supplementary material for: A Quorum Sensing-Disrupting Brominated Thiophenone with a Promising Therapeutic Potential to Treat Luminescent Vibriosis
Source: PLoS One. 2012 Jul 25;7(7):e41788. doi: 10.1371/journal.pone.0041788 (PMC3404956; doi:10.1371/journal.pone.0041788)
Supplement: Information S1 — Synthesis of (Z)-4-((5-(bromomethylene)-2-oxo-2,5-dihydrothiophen-3-yl)methoxy)-4-oxobutanoic acid (TF310). (DOC) [file pone.0041788.s001.doc]

**A quorum sensing-disrupting brominated thiophenone with a promising therapeutic potential to treat luminescent vibriosis**

Tom Defoirdt, Tore Benneche, Gilles Brackman, Tom Coenye, Patrick Sorgeloos, and Anne Aamdal Scheie

**Supporting information: Synthesis of (Z)-4-((5-(bromomethylene)-2-oxo-2,5-dihydrothiophen-3-yl)methoxy)-4-oxobutanoic acid (TF310).**

Ethyldiisopropylamine (155 mg, 1.2 mmol) and DMAP (catalytic amount, ~10 mg) was dissolved in dichloromethane (2 mL) and added to a solution of succinic anhydride (120 mg, 1.2 mmol) and (*Z*)-5-(bromomethylene)-3-(hydroxymethyl)thiophen-2(5*H*)-one^1^ (0.22 g, 1.0 mmol) in at room temperature. The reaction mixture was stirred for 30 minutes, diluted with dichloromethane (25 mL) and washed with water (3x5 mL). The combined aqueous phases were extracted with ether (2x10 mL). The combined organic phases were dried (MgSO_4_), filtrated and the solvents evaporated off. The residue was dissolved in a small amount of THF and ether (1:2), and the product was precipitated by addition of pentane. The solution was filtered, leaving a yellow solid.

Yield: 220mg (69%)(yellow solid)

Melting point: 79 – 80 ºC

^1^H-NMR: 10.8(bs, 1H), 7.41(s, 1H), 7.12(s, 1H), 4.87(s, 2H), 2.73-2.63(m, 4H)

^13^C-NMR: 191.4, 178.2, 171.6, 144.0, 142.6, 140.2, 113.3, 58.5, 28.8, 28.6

MS(EI): 322(6.0), 320(5.9), 222(83.9), 220(82.8), 141(39.3), 101(100)

HR-MS(EI): calculated for C_10_H_9_BrO_5_S: 319.9354

found for C_10_H_9_BrO_5_S: 319.9359

IR: 3317, 3058, 2938, 1733, 1705, 1667, 1610, 1582
